# Poster Session II - A276 COMPARISON OF PATIENT RESPONSE TO FIRST VERSUS SECOND-LINE INFLIXIMAB IN MODERATE-TO-SEVERE ULCERATIVE COLITIS: A RETROSPECTIVE STUDY

**DOI:** 10.1093/jcag/gwaf042.276

**Published:** 2026-02-13

**Authors:** C Galts, S Anvari, M Haq, K Grossman, D Borovsky, A Wen, E Wong, H R Tran, A Albassam, S Halder, J Marshall, N Narula

**Affiliations:** Gastroenterology, McMaster University, Hamilton, ON, Canada; McMaster University Faculty of Health Sciences, Hamilton, ON, Canada; McMaster University Faculty of Health Sciences, Hamilton, ON, Canada; Gastroenterology, McMaster University, Hamilton, ON, Canada; McMaster University Faculty of Health Sciences, Hamilton, ON, Canada; McMaster University, Hamilton, ON, Canada; Gastroenterology, McMaster University, Hamilton, ON, Canada; Gastroenterology, McMaster University, Hamilton, ON, Canada; Gastroenterology, McMaster University, Hamilton, ON, Canada; Gastroenterology, McMaster University, Hamilton, ON, Canada; Gastroenterology, McMaster University, Hamilton, ON, Canada; Gastroenterology, McMaster University, Hamilton, ON, Canada

## Abstract

**Background:**

Infliximab has remained a cornerstone in the management of moderate and/or severe ulcerative colitis (UC), however its outcomes after other advanced therapy failure has not been evaluated comprehensively

**Aims:**

This study evaluates infliximab’s second-line effectiveness, identifies predictors of response, and compares outcomes to its first-line use.

**Methods:**

A retrospective cohort study of adults with moderate or severe UC and treated with infliximab was identified at a tertiary care center between 2016-2024. Those patients were then categorized by line of therapy. Multivariable logistic regression was used to assess predictors of one-year clinical remission

**Results:**

Among the 225 patients who met the study criteria, 143 (63.6%) received infliximab as first-line and 82 (36.4%) as second-line or subsequent therapy (Table 1). Clinical response at one year was significantly lower in second-line users (odds ratio 0.53, 95%CI 0.28–0.99, p = 0.049), and remained significantly lower after adjustment for biosimilar and concurrent steroid use. Other outcomes including one-year clinical remission, endoscopic improvement or remission were not significant (OR 0.69 (95% CI 0.44-1.08), p = 0.102; OR 0.84 (95% CI 0.50-1.43), p = 0.522; OR 0.79 (95% CI 0.46-1.34), p = 0.378; respectively). There was significantly more dose escalation of infliximab within one year (p = 0.049) among patients who had prior advanced therapy, as well as more discontinuation of infliximab within one year (p = 0.045), compared with first-line infliximab treated patients (Figure 1).

**Conclusions:**

Infliximab continues to demonstrate high rate of clinical and endoscopic response, however its clinical response is significantly lower than in patients who received infliximab first-line. These findings support its continued use while highlighting the need for optimized patient selection and treatment strategies in therapy-exposed populations.

A276 Table 1: Baseline characteristics

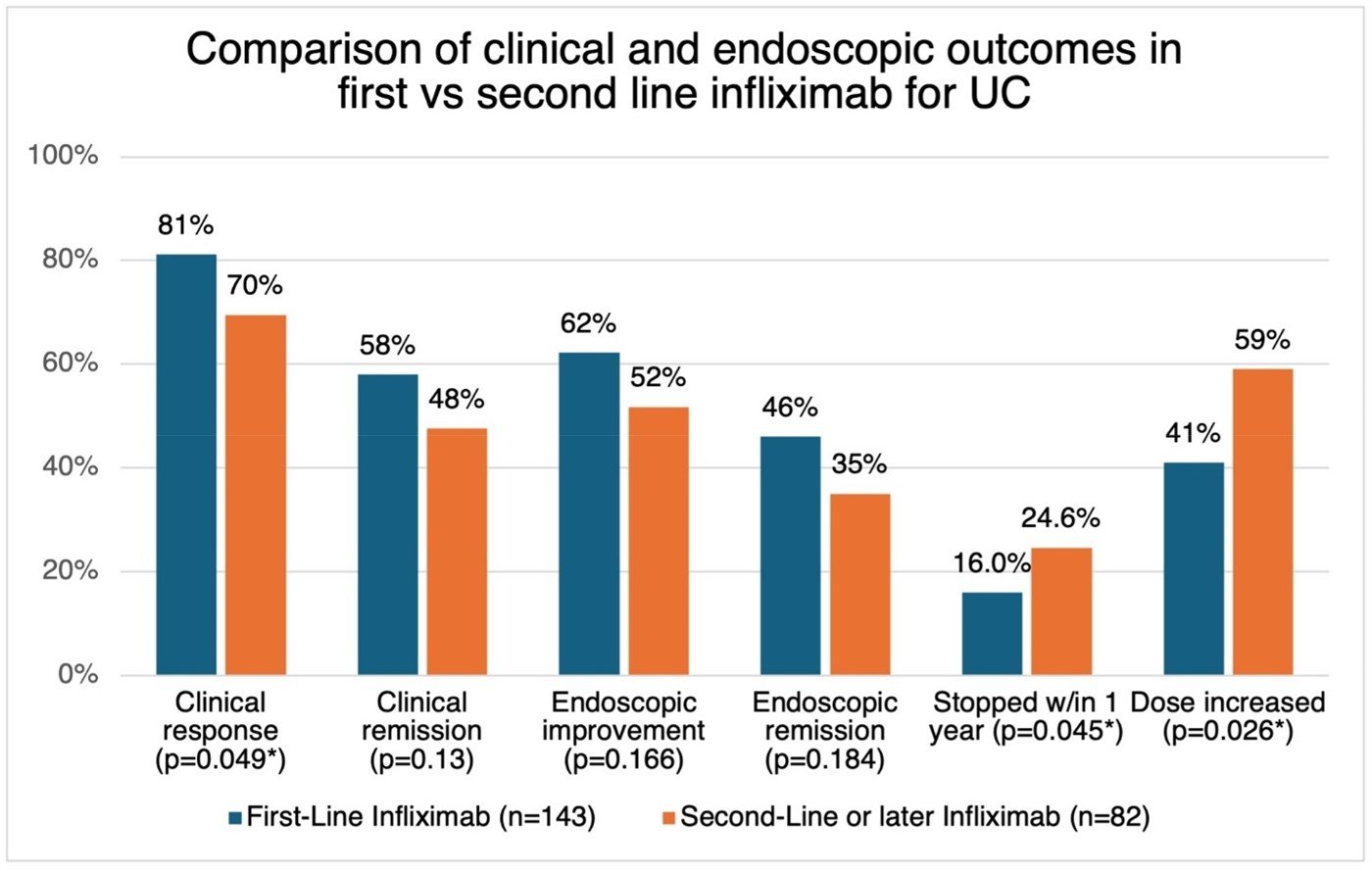

**Funding Agencies:**

None

**Table:** 

Variable	Overall (n = 225)	First-line infliximab (n = 143)	Second-line infliximab + (n = 82)	p-value
Age at diagnosis, mean (SD)	31.3 (14.4)	32.0 (14.4)	30.1 (14.5)	0.345
Female, n (%)	103 (45.8)	65 (45.5)	38 (46.3)	0.898
Disease extent, n (%)				0.704
Proctitis	24 (10.8)	17/142 (12.0)	7/81 (8.6)	
Left-sided	72 (32.3)	47/142 (33.1)	25/81 (30.8)	
Pancolitis	127 (57.0)	78/142 (54.9)	49/81 (60.5)	
Smoker, n (%)	22 (10.0)	10/140 (7.1)	12/80(15.0)	0.086
Infliximab started as in-patient, n (%)	64 (28.4)	51 (35.7)	13 (15.9)	0.002
Concurrent azathioprine, 6-MP or methotrexate while on infliximab, n (%)	78 (34.7)	53 (37.1)	25 (30.5)	0.315
Concurrent steroid use > 10mg/d at induction, n (%)	57 (25.3)	33 (23.1)	24 (29.3)	0.308

